# The global nuclear order and the crisis of the nuclear non-proliferation regime: Taking stock and moving forward

**DOI:** 10.1007/s42597-021-00066-0

**Published:** 2022-01-19

**Authors:** Jana Baldus, Harald Müller, Carmen Wunderlich

**Affiliations:** 1 Hessische Stiftung Friedens- und Konfliktforschung, Frankfurt, Germany; 2 Lehrstuhl für Internationale Beziehungen und Entwicklungspolitik, Universität Duisburg-Essen, Duisburg, Germany

**Keywords:** Nuclear order, NPT, Nuclear proliferation, Nuclear disarmament, TPNW, Nukleare Ordnung, NPT, Nukleare Weiterverbreitung, Nukleare Abrüstung, TPNW

## Abstract

The Nuclear Non-Proliferation Treaty (NPT) is a central element of the global nuclear order, the primary goal of which is to prevent nuclear war. But this understanding is being threatened by a number of developments. Frustration about the lack of nuclear disarmament and concerns about humanitarian consequences of nuclear weapons led to the negotiation of the Treaty on the Prohibition of Nuclear Weapons (TPNW). The new treaty has further exposed existing fault lines within the NPT and exacerbated unresolved conflicts over the proper approach to disarmament and the weighting of the NPT pillars. Currently, disagreements over the compatibility of the two treaties and the approach to the TPNW in particular divide the membership of the NPT. At the same time, real proliferation cases test the regime’s ability to act, as norm enforcement is regularly hampered by interference from the great powers. These developments—the absence of genuine disarmament, disputes among NPT members, competition between the TPNW and the NPT, and actual nuclear proliferation—are part of a comprehensive crisis and destabilization of the non-proliferation regime. We argue that three developments are necessary to restabilize the regime: a cooperative resolution of the power struggles among the major powers; a depolarization of the inter-group divisions in the NPT; and a return to the principle of nuclear war prevention as a common maxim for action.

## Abstract (de)

Der Atomwaffensperrvertrag ist ein zentrales Element der globalen nuklearen Ordnung, deren primäres Ziel es ist, einen Atomkrieg zu verhindern. Doch dieses Einvernehmen ist durch eine Reihe von Entwicklungen bedroht. Die Frustration über unzureichenden Fortschritt bei nuklearer Abrüstung und die Sorge über die humanitären Folgen von Atomwaffen haben zur Aushandlung des Atomwaffenverbotsvertrags geführt. Der neue Vertrag hat bestehende Konfliktlinien innerhalb des NVV offengelegt und ungelöste Konflikte über den richtigen Ansatz zur Abrüstung und die Gewichtung der Pfeiler des NVVs verschärft. Gegenwärtig spaltet insbesondere die Uneinigkeit über die Vereinbarkeit der beiden Verträge und über den Umgang mit dem TPNW die Gemeinschaft des NVV. Gleichzeitig stellen konkrete Proliferationsfälle die Handlungsfähigkeit des Regimes auf die Probe, da die Durchsetzung der Normen regelmäßig durch die Einmischung von Großmächten behindert wird. Diese Entwicklungen – das Fehlen echter Abrüstung, Kontroversen zwischen den NVV-Mitgliedern, die Konkurrenz zwischen TPNW und NVV und tatsächliche nukleare Proliferation – sind Teil einer umfassenden Krise und Destabilisierung des Nichtverbreitungsregimes. Wir argumentieren, dass drei Entwicklungen notwendig sind, um das Regime zu restabilisieren: eine kooperative Beilegung der Machtkämpfe zwischen den Großmächten; eine Depolarisierung der Spaltungen zwischen den Gruppen im NVV; und eine Rückkehr zum Prinzip der nuklearen Kriegsverhütung als gemeinsame Handlungsmaxime.

## Introduction

In 1995, the Review and Extension Conference of the Treaty on the Non-Proliferation of Nuclear Weapons (NPT) decided, without a vote, to extend the treaty indefinitely, after the initial duration of 25 years ended. Optimism about the non-proliferation regime abounded. The 2000 Review Conference (RevCon) resulted in a substantial consensus on all three pillars of the NPT—non-proliferation, peaceful uses of nuclear energy, and, most significantly, nuclear disarmament, including 13 practical steps. Modest, but steady progress of the regime seemed foreordained.

Twenty years later, the situation has deteriorated dramatically. The members of the treaty are more divided than ever (Müller 2017). Nuclear arms control and disarmament are in an agonizing process of seemingly inevitable dismantlement. This prompted a majority of States Parties to conclude a new treaty, the Treaty on the Prohibition of Nuclear Weapons (TPNW), which entered into force in January 2021 after 50 ratifications had been reached. The background and main argument for negotiating the TPNW were the humanitarian consequences that could result from continued nuclear rivalry. But the TPNW also aimed to challenge entrenched power relations and give a voice to the resentments and concerns of non-nuclear weapon states (NNWS) and civil society actors about the lack of nuclear disarmament. In addition to the great enthusiasm it has generated, the TPNW has also caused some turmoil and further exposed existing fault lines within the NPT. And indeed, adversarial policies concerning this treaty could, in the worst case, lead to a break-up of the NPT community and to competition between two treaties in the same policy field. Furthermore, nuclear proliferation has taken place; India and Pakistan tested nuclear weapons in 1998 and have steadily developed nuclear arsenals ever since; North Korea has tested nuclear weapons, and Iran made progress toward acquiring nuclear capability, at least until 2003. The Iranian program might be revived if the Joint Comprehensive Plan of Action (JCPoA) of 2015, negotiated between Iran, France, Germany, the United Kingdom (UK), the European Union (EU), the United States, China, and Russia ends up disintegrating as a result of US withdrawal in 2019 (Tannenwald 2018). Whether efforts to prevent the collapse of the agreement by the Biden administration will be successful is not yet foreseeable at the time of writing given the difficult negotiation process.

The absence of meaningful disarmament, disputes within the NPT membership, competition between the NPT and the TPNW, and real nuclear proliferation mark the four aspects of a comprehensive regime crisis.^1^ The widely discussed crisis of the NPT is both an exceptional situation arising from specific moments of contestation and an intrinsic and latent condition of the non-proliferation regime; it is as much the expression of a stasis or even a decline of what had already been achieved as of new impetus and new agency. This article aims to reflect on the crisis of the global nuclear order in terms of these four dimensions. In so doing it provides a comprehensive overview of the fundamental conflicts underlying the crisis, which are particularly evident in the disputes within the NPT community and which are closely linked to the emergence of the TPNW. The focus is on both the internal and external embeddedness of the crisis. Thus, attention is paid not only to its structural foundations, such as the intra-regime conflicts that have always been part of the NPT (Miller 2012), but also on how the crisis is linked to current geostrategic affairs, the failure of further arms control efforts, and proliferation crises.

The article shows that these crisis dimensions are closely intertwined, even though they are often considered separately: if the crisis intensifies in one area, this has a direct impact on the other dimensions. It follows that only a comprehensive response to the regime crisis, one which considers all dimensions, can stabilize and sustain the global nuclear order. In light of this, the article proceeds as follows: first, we analyze the place of the NPT in the global nuclear order. We then address the various crisis dimensions: the changing geostrategic context and the breakdown of nuclear arms control; intra-regime conflicts; the relationship between the TPNW and the NPT; and current proliferation crises. Based on this analysis, we develop a series of suggestions for positive development of the regime.

## The place of the nuclear non-proliferation regime in the global nuclear order

The term “order” requires a caveat. Order invokes the idea of something purposeful and human-made. This association is only half true. What we call global order is the vector of the interplay of actions of quite a large of number actors—including both states and non-state entities. Some actors may indeed aim at some form of *order*; but what they envisage may be no more than their own rule over the rest; or something that gives them security with little regard to the security of everybody else; or a system shaped by an unifying ideology which others dislike, etc.. There may be actors who try to set up a cooperative order in the common interest, or others who at least want to build an order of compromise among many differing interests. Today, we can find evidence of all these notions of order, and the existing order is the result of this panoply of actions pulling and pushing in many different directions.

Whether we look at today’s world from a moralistic or a rationalistic perspective, global order is subjected to the imperative of preventing nuclear war. All the objectives of the many different actors will become irrelevant if this ultimate human catastrophe ever becomes reality (Lodgaard 2019). It took more than 15 years in the nuclear age and one confrontation which almost resulted in nuclear escalation, the Cuban missile crisis of 1962, for the two nuclear superpowers to understand this imperative. From the early mid-sixties on, they began to construct the building blocks of an order intended to help prevent nuclear war, occasionally assisted and complemented by their allies, neutral and non-aligned states, and closely observed and guided (frequently to no avail) by an increasing number of non-governmental actors.

We can distinguish between four practical operative elements of this order:^2^ (a) political and military crisis prevention and crisis management, for example the famous “red telephone”, agreements to prevent nuclear war and dangerous military accidents at sea, tolerance for satellite observation, or missile launch notifications; (b) bilateral and multilateral nuclear arms control and disarmament measures such as the START Treaties or the Comprehensive Test Ban Treaty (CTBT); (c) direct contact and talks between the nuclear weapon states (NWS), for example in the context of P‑5 consultations in the margins of the United Nations Security Council (UNSC) or during the specific nuclear weapons talks at expert level in which the US, Russia, the UK, France, and China have engaged since 2009; (d) the fourth cornerstone is the nuclear non-proliferation regime with the NPT at its core.

Thus, the NPT has been a key part of the efforts to avoid a nuclear Armageddon. Now though, it seems as though the actors have lost sight of this central objective of the treaty, it is something that has disappeared from their collective memory. This amnesia is strange, as the NPT’s preamble starts with a paragraph characterizing nuclear war as an immense catastrophe for humankind.^3^ The preamble continues with the unambiguous imperative to prevent this catastrophe—proliferation must be prevented precisely because more nuclear weapons possessors means a higher probability that nuclear war will occur.

The NPT is the only existing international legal instrument which obliges its five nuclear weapon states parties to pursue complete nuclear disarmament. This undertaking has two aspects: First, it represents a quid pro quo for the vast majority’s voluntary renunciation of the most powerful weapon of the time. Second, the commitment of the NWS to disarm is another contribution to the endeavor to prevent nuclear war: when there are no more nuclear weapons, they can no longer be used. The majority of the non-nuclear weapon states (NNWS) regard nuclear disarmament as the ultimate guarantee that nuclear war will not occur, the core objective of the NPT.

When the NPT was initiated, negotiated, and concluded, the achievement of the above objective was being jeopardized by two processes: First, an increasing number of states possessed basic technical capabilities which could result, sooner or later, in the development and production of nuclear weapons. Second, the nuclear superpowers, the US and the Soviet Union, were involved in a nuclear arms race. The NPT addressed the first problem, bilateral arms control dealt with the second one. The emerging nuclear order was thus characterized by a threefold self-constraint: (a) The NNWS renounced nuclear weapons; (b) the NWS imposed limits on their nuclear competition and tried to manage their geopolitical rivalry so as to avoid deadly crises prone to escalation; (c) the NWS agreed to a legal obligation to accept further limitations on their nuclear arsenal and eventually their complete elimination.

William Walker has called this order an “enlightenment project” (Walker 2012): understanding the risks of self-devastation prohibited an unlimited pursuit of power preponderance under conditions of interdependent security. The self-restraint embodied in the various renunciations and limitations and the resulting order were products of political reason.

## Taking stock: The nuclear non-proliferation regime under strain

### The changing geostrategic context and the breakdown of nuclear arms control

In the decade from 1986 to 1996, the *enlightenment project *seemed to prevail. Geopolitical conflicts of the Cold War were solved. A sequence of nuclear and non-nuclear arms control and disarmament measures of unprecedented density ended the arms race. The Soviet nuclear legacy did not lead to proliferation: Ukraine, Belarus, and Kazakhstan agreed to transfer the nuclear weapons on their territories to Russia and to accede to the NPT and the NNWS, as did all the other *fission products* of the former Soviet Union and Yugoslavia. Important holdouts such as Spain, Argentina, Brazil, Algeria, Saudi Arabia, and most other Arab states decided to join the NPT. South Africa dismantled its nuclear weapons and related facilities and became party to the NPT, and other African states, which had remained outside the treaty because South Africa had, soon followed suit. China and France, who had originally refused to sign the treaty, ratified the NPT as well. It was in the context of this wave of real disarmament—and the expectation of more—and the apparent irresistibility of the NPT that a conference of parties decided to make the duration of the treaty indefinite in 1995.

The euphoria did not last, however. Rivalry between the US and Russia reemerged in Europe and extended to the Middle East, Northern Africa, the Caucasus, and Central Asia. China’s rise to power added another contender, and exacerbated US-Chinese rivalry in East and Southeast Asia, as well as in South Asia where India had become more open to an entente with the US (Walker 2012). China’s geo-economic policy developed into another strand of competition for power and influence.

Inevitably, this plurilateral power rivalry had military aspects. As the competitors were NWS, nuclear weapons regained prominence in national doctrines and strategies. The “cessation of the nuclear arms race” as called for in NPT Art. VI came to an end. Nuclear arms control and disarmament grinded to a halt and was even reversed by the termination of the ABM-; INF- and START II-Treaties. The specter of nuclear war hung over the horizon once again (Tannenwald 2018; Akit 2020).

These developments have a negative impact on non-proliferation in three ways: First, great power rivalry tends to make regional conflicts more acute. The security environment of the NNWS becomes less favorable, and local and regional rivalries acquire global importance, making smaller states players in the great powers game. As the powers tend to favor one side in these conflicts, they become a threat to the other side. The fact that other great powers offer to serve as protectors is only minor consolation: such promises may quickly become empty, and sovereignty may suffer from increasing dependency. This situation creates an inherent motivation for countries to consider going nuclear: nationally owned nuclear weapons can be perceived as a useful deterrent, a symbol of independence, and a handy instrument for power politics.

Second, the willingness of the great powers to reign in reticent allies for the sake of non-proliferation declines as the great power competition increases. Priorities change: non-proliferation does not become irrelevant but is subordinated to geopolitical considerations. For example, the US has almost single-handedly put an end to the boycott against nuclear trade with India in the Nuclear Suppliers Group (Potter 2005; Bajoria and Pan 2010) and is ready to enter major nuclear cooperation with its allies in the Persian Gulf (Gilinsky and Sokolski 2020). China, for its part, has no qualms about engaging in nuclear trade with Pakistan. The non-proliferation norm has become a tool of political opportunism: it is invoked when it serves the great power’s geopolitical interests and ignored when it does not. This practice is destructive in the context of a norm that can only function if it is adhered to universally.

Third, the ongoing competition in nuclear arms, accompanied by the dismantlement of the arms control and disarmament edifice, increases the tensions within the NPT membership. The legal commitment of the NWS to nuclear disarmament is part and parcel of the NPT structure. Disregarding this obligation increases the virulent dissatisfaction among NNWS parties about the growth of inequality of duties and rights as a result of the inequality of compliance. Intra-regime conflicts which have existed from the beginning (Müller and Wunderlich 2018) are thus more bitter than ever.

### Intra-regime conflicts

From the outset, the NPT’s discriminatory structure has perpetuated intra-regime conflicts. The *legalized* dichotomy between nuclear weapon states and non-nuclear weapon states, and the resulting uneven distribution of responsibilities and obligations under the treaty, is the main reason the legitimacy of the NPT is eroding. The NPT’s legitimacy was always “based on a principled justification of a temporary inequality, and a balancing of norms, rights, and obligations designed to limit, and ultimately eliminate, the fundamental discrimination of the regime” (Tannenwald 2013, p. 314). Yet, by not credibly fulfilling their disarmament obligation, the NWS have caused cracks in the fragile system. It is not by chance that the political conflicts within the NPT have for some time now focused largely on questions related to nuclear disarmament, and only to a lesser degree on the situation in the Middle East or on cases of non-compliance with the non-proliferation norm. Rather, the current crisis is a result of precisely this denial of the special responsibility of the NWS, a changed (geo-)political setting in which (nuclear) confrontation has become the new normal, and deeply rooted conflicts that have been smoldering since day one of the treaty.

Apart from the vertical division of the treaty (nuclear weapon states vs. non-nuclear weapon states), the horizontal structure of the NPT—the three pillars of non-proliferation, disarmament, and peaceful uses of nuclear energy—has long given rise to serious disputes. Historically, the three pillars must be understood as equal and symmetrical; they cannot be separated from one other and should be understood as mutually reinforcing (Joyner 2011). Although the initial US-Soviet draft for the NPT envisioned a treaty with the sole purpose of preventing further nuclear proliferation, the negotiations with the NNWS soon resulted in a broader mission for the NPT (Shaker 1980). The grand bargain between the NWS and the NNWS called for tangible compensation for the burden of non-proliferation measures and encroachment on sovereignty rights (Popp 2017). (It is indeed hard to imagine a non-proliferation treaty in which the majority of states would have been willing to forsake a nuclear option or accept the nuclear armament of some states without any concession from them.) But the negotiation history of the NPT also reflects the understanding that it is the interplay of the three pillars that supports the fulfilment of the NPT’s main objective, namely the prevention of nuclear war (Müller 2019). Both peaceful uses of nuclear energy and support for non-proliferation enable nuclear disarmament and are thus vital building blocks in the creation of a nuclear-weapon-free world. Progress toward disarmament is important as it “strengthens the legitimacy of the regime by creating the expectation that the special rights of the nuclear weapon states will end at some point in the future” (Rathbun 2006, p. 233). As such, the legitimacy of the whole regime as well as further non-proliferation measures depends, to some degree, on the disarmament record of the NWS.

The NWS regularly stress that the main objective of the NPT is, and indeed should be, the prevention of nuclear proliferation. The NNWS’ support for more or less intrusive non-proliferation measures is, in turn, interpreted as a condition for technical cooperation; nuclear disarmament, on the other hand, is seen only as a secondary obligation that is dependent on (somewhat vague) *favorable* conditions (Ford 2018). While many NNWS emphasize their support for equal weighting of each of the three pillars, they maintain that further action in the field of non-proliferation should be dependent on progress in nuclear disarmament and cooperation on peaceful uses of nuclear energy. In the past, countries belonging to the Non-Aligned Movement (NAM) have gone so far as to block any progress in nuclear non-proliferation, which could entail more obligations for the NNWS, as long as the NWS fail to advance nuclear disarmament (Müller 2019). In both cases, the much-cited pillar structure of the NPT is abandoned in favor of a quasi-hierarchical reading of the treaty: in the first case, it is the non-proliferation norm that is elevated above measures for technical cooperation and nuclear disarmament; in the second case, nuclear disarmament should precede further action on non-proliferation.

Discussions about the interpretation of the NPT’s most important norms are as old as the treaty itself. In addition to the relative weighting of the NPT’s pillars, opinions also differ on the readings of Article VI and the preamble, the interpretation and scope of the “inalienable right” to the peaceful uses of nuclear energy (Article IV), the handling of cases of nuclear proliferation and non-compliance (see below), and the approach towards a WMD-free zone in the Middle East. The most controversial questions are whether the nuclear disarmament clause is indeed an obligation and whether the ultimate goal of the treaty is to foster progress in nuclear disarmament or to prevent nuclear proliferation (Miller 2012). Closely related is the question of whether the NPT’s provisions grant indefinite legitimacy to the unequal structure of the treaty, i.e. are to be understood as accepting that a few states can indefinitely maintain their privileges vis-á-vis the vast majority of states (Walker 2012). The behavior of the NWS suggests that this precisely is what they think. Yet, the adoption of the TPNW has shown that most NNWS are no longer willing to accept this logic, and their position finds support in the authoritative advisory opinion of the International Court of Justice (1996) (ICJ 1996). As a result of the latitude in the interpretation of Art. VI, the NWS have in the past depicted their disarmament record quite generously. The US and Russia regularly point to the impressive cuts in their nuclear arsenals since the INF Treaty of 1987. The NWS have also made some progress regarding transparency on their nuclear arsenals. In most cases, however, they reject further disarmament measures, citing an unfavorable security environment (Meyer 2019). They have long regarded the disarmament issue as their turf, and something in which the NNWS do not have any influence. As a consequence, many NNWS have been increasingly critical (and vocal in that criticism) of what they perceived as a lack of willingness to meet one of the core obligations of the treaty (Müller and Wunderlich 2020).

The only controversy as heated as the issue of disarmament concerns the universalization of the treaty, a dispute which is particularly evident in the contestation regarding the establishment of a WMD-free zone in the Middle East. The discussions on the WMD-free zone are a peculiar legacy of the indefinite extension of the NPT in 1995 (although the debate itself dates back to the 1970s). During the negotiations on the indefinite extension of the NPT, Egypt used the NWS’ desire for the Arab Union to endorse a final agreement as leverage to achieve a quid pro quo on the nuclear asymmetry in the Middle East (Miller 2012). As a result, the depositary states of the NPT (US, Russia, and the UK) felt compelled to draft a resolution committing to making progress toward a WMD-free zone in the Middle East (Müller 2011). While there has been agreement on the general desirability of a WMD-free zone in the Middle East for a long time, there is no consensus on how the “Middle East resolution” should be implemented. The debate is dominated by the juxtaposition of “peace process vs. disarmament,” which, over time, has developed into a stalemate predominantly between two sides: the Arab Union on one side (“disarmament first”), Israel with US support on the other (“peace process first”) (Kubbig and Weidlich 2015). Both in 2005 and 2015, the general approach to the 1995 Middle East Resolution was a decisive factor in the failure to come to an agreement on other issues. Without the deadlock between the two opposing fronts—on the one hand, the Arab League with Egypt as the “political director”; on the other, the United States as a staunch supporter of Israeli interests in the NPT context—compromises on nuclear disarmament might have been possible (Potter 2016). 2019 saw new momentum on the matter when all regional stakeholders, with the exception of Israel, convened at a conference tasked with preparing the ground for establishing a WMD-free zone in the Middle East, which resulted in a political declaration to continue the process. This change of fora plus the significant realignments in the Middle East have likely eased the pressure that the controversial issue had placed on previous RevCons (Bino 2020). Furthermore, thanks to the TPNW, the issue has recently receded into the background somewhat. Nevertheless, the handling of the Middle East Resolution, which has almost become something akin to the “fourth pillar” of the NPT, may once again be decisive for the outcome of the next RevCon.

These issues, which have caused a great deal of controversy in the past, must now be seen against the backdrop of two opposing trends: on the one hand, the formation of a broad civil society and state front which aims to delegitimize nuclear weapons altogether; on the other, the burgeoning great power conflict between the US, Russia, and China, coupled with ongoing modernization of their nuclear arsenals (Erästö and Cronberg 2018). Even after modest progress in nuclear disarmament and the formulation of new obligations for the NWS under the NPT, the regime retains its highly unequal structure. While the Outcome Documents from 2000 and 2010 specified the NWS’ obligations and reduced the imbalance of rights and obligations between NWS and NNWS, the dissatisfaction among NNWS has only grown in recent years, particularly because the NWS have largely ignored the political commitments they made in agreeing to these two documents. The changed (geo-)political context, which is something the nuclear armed great powers are primarily responsible for, as well as the dismissive attitude of US Republicans toward arms control, have further diminished the NWS’ readiness for nuclear disarmament, and even prompted the US and Russia to begin a roll-back of what had been achieved (Gibbons 2019).

So far, the NWS have mainly blamed the TPNW and its supporters for the dire position in which the NPT has been put (see below). The NWS’ narrative ignores that the growing rivalry between the major powers makes success in nuclear disarmament or arms control highly unlikely. The nuclear powers’ constant denial of their disarmament obligations seriously damages the NPT’s *grand bargain*: The self-restraint of the NNWS (perpetuated by the indefinite extension of the NPT in 1995) is no longer balanced by the compliance of the NWS with their disarmament obligations. This reinforces the justice issues that are at the heart of the treaty and that have existed since its inception (Müller 2010a, 2019; Tannenwald 2018). The unequal structure of the NPT has created a dividing line between nuclear weapon states (plus allies) on the one hand, and most NNWS, on the other. However, an important point is often lost in the current debate: Neither the P5 and NATO, nor the NNWS, or even the NAM are homogenous groups. The position lines in the NPT are not set in stone; they are permeable and have also occasionally shifted. Currently, most group lines are portrayed as a given. In this scenario, there is no doubt which states should support the TPNW and which should contest it. As a result, states that were always renowned for their ability to mediate between the poles have either been marginalized (Switzerland, Sweden) or put under pressure to show solidarity, thus making them ineffective as mediators (Australia, Canada, Germany, South Africa, New Zealand). New attempts at bridge-building are in constant danger of losing credibility on both sides. Even on the occasion of the 50th anniversary of the NPT, the polarization among NPT membership was hard to conceal. After lengthy negotiations in the UN Security Council, the only outcome was a generic press statement that acknowledged the NPT’s contributions to international security.^4^

As a result of the current focus on the TPNW, thematic areas within the NPT that would usually be more conducive to compromise, such as peaceful use of nuclear energy, are either thrust aside or run the risk of being politicized as well. It is against this backdrop that Gustavo Zlauvinen, the President-Designate of the 2022 RevCon, has announced that the conference will be tailored to the issue of peaceful use. While this strategy could help to find a common basis for discussion, it is unlikely that the conflicts over nuclear disarmament and the TPNW can be suppressed for long. The conflict over the nuclear-weapon-free zone in the Middle East is also likely to retain its significance and role as a possible showstopper at NPT review conferences, depending on how Egypt, the “political director” of the Arab League, plays its cards. In the current situation, it is questionable whether the NPT community can use the additional time, gained due to the postponement of the 2020 RevCon because of the Covid-19 pandemic, to resolve their conflicts. What could bring them together is perhaps a renewed commitment to the NPT’s founding mission—the prevention of nuclear war—, an unequivocal commitment to the mutually supportive relationship of the treaty’s three pillars, an agreement on some tangible practical disarmament steps,^5^ and a modus vivendi—tacit or explicit—to deal with the TPNW.

### New kid on the block: the treaty on the prohibition of nuclear weapons

Internal regime conflicts have eventually divided NPT parties even on the legal plane. Frustrated with the refusal of several NWS in 2005 to recognize the political validity of previous agreements on nuclear disarmament, some disarmament supporters—state and non-governmental—began to deliberate on a new path toward nuclear disarmament: the development of a legal instrument to prohibit nuclear weapons outside the traditional venues where nuclear disarmament was considered, namely the NPT Reviews and the Geneva Conference on Disarmament. They rallied around concerns about the humanitarian consequences of nuclear explosions. New scientific insights into both these consequences and the increased probability that nuclear explosions or even nuclear war could actually happen, necessitated, in their view, prompt initiatives to eliminate nuclear weapons, just as had been the case with the other weapon types in the *weapons of mass destruction* category, chemical and biological weapons (Borrie 2014).

The humanitarian aspect was successfully inserted into the final document of the 2010 NPT RevCon. In 2013/14, three international conferences were held to present and debate the humanitarian aspects of nuclear catastrophes in great detail based on scientific studies. The failed 2015 RevCon prompted the translation of the new concept into practice. A majority in the United Nations General Assembly (UNGA) established an Open-Ended Working Group (OEWG) to discuss new paths to nuclear disarmament, and, on the basis of the OEWG’s report, created a negotiation body to develop a legal instrument for prohibiting nuclear weapons. The negotiations were completed by July 7, 2017, after just two rounds of talks, and the UNGA supported the opening of the TPNW for signature on September 20, 2017 (Mukhatzhanova 2017; Potter 2017). On October 24, 2020, the TPNW reached its 50th ratification and, as a result, entered into force 90 days later (January 22, 2021).

The NWS and their allies boycotted the negotiations (with the exception of the Netherlands) and have almost categorically rejected the TPNW and any discussions on it (see, e.g. UK Mission the UN 2017). The opponents of the new treaty base their opposition on three main arguments^6^: the alleged legal incompatibility of the NPT and the TPNW, the supposed undermining of the NPT by the TPNW, and the purported ineffectiveness of the TPNW (see, e.g. Ford 2017; Highsmith and Stewart 2018; Mount and Nephew 2017; NATO 2020).

The relationship between the TPNW and the NPT and the role of the former in nuclear disarmament can be summarized as follows. First, the TPNW constitutes a strong normative statement by a large number of NNWS. It demonstrates their determination to move toward a world without nuclear weapons, and their profound dissatisfaction with the current state of nuclear disarmament. It expresses their moral condemnation of nuclear weapons and nuclear deterrence. It also manifests their desire not to regard nuclear disarmament as the *chasse gardée* of the NWS, but to participate in the process and take the initiative, if necessary, without the NWS.

Second, the TPNW alone will not lead to nuclear disarmament without the cooperation and active involvement of the NWS. The concept that nuclear disarmament will be achieved by the holdouts successively acceding to the TPNW until all states are parties is a pipe dream. The mutual security conflicts and ensuing distrust among the great powers and the technical complexities of their deterrence systems require them to engage in a series of bilateral and plurilateral negotiations in order to move nuclear disarmament forward. The TPNW constitutes a normative imperative and a tool to campaign for exerting pressure on reluctant states, no more, no less.

Third, the TPNW does not provide sufficient basis to grant security in a world without nuclear weapons. Like the NPT, it does not prohibit nuclear weapons research, nor does it contain any specific rules for nuclear related exports. While the TPNW requires NWS who want to become parties to negotiate appropriate verification measures (Shea 2020), it accepts the present verification system of its NNWS members which are already parties to the NPT. This means that states which do not have an Additional Protocol (AP) in force would not be obliged to accept verification measures that could detect clandestine activities; the old NPT verification system is not capable of exposing what states want to conceal. This is too little to provide security in a nuclear-weapon-free world. The TPNW does not set out measures for addressing non-compliance and enforcement, which would be essential to create confidence in the security of a nuclear-weapon-free world (Müller 2010b).

Fourth, the TPNW is fully compatible with the NPT (Hajnoczi 2020; Soares de Macedo 2018). It contains all prohibitions of the NPT and indeed goes further; the only thing missing, as mentioned, is export control, which is covered only in a general way by the ban on supporting nuclear weapons activities. No undertaking in the TPNW contradicts undertakings in the NPT. The weaknesses of the TPNW (nuclear weapons research, verification, enforcement) are also weaknesses of the NPT. The accusation that the TPNW threatens to “supersede” the NPT is without substance as has been amply demonstrated (Müller and Wunderlich 2020; Deutscher Bundestag, Wissenschaftliche Dienste 2021).

Fifth, the TPNW does not split the NPT community, as its opponents maintain (see, e.g. Ford 2017), but is clearly the product of a split that has existed from the birth of the NPT and has been exacerbated by the demise of nuclear arms control and disarmament, combined with the NWS disregarding the agreed measures adopted by previous NPT RevCons (Meyer and Sauer 2018). The impact of the TPNW’s existence is entirely in the hands of the two opposing camps on this issue: the promoters of and parties to the ban treaty on the one hand, and its opponents, the NWS and their allies, on the other. Whether they opt for mutual accommodation, compromise, and the pursuit of ways forward or, on the contrary, for an endless confrontation with no way out, will decide on the future of both disarmament and non-proliferation.

So far, signs are mixed. The preparatory meetings for the next NPT RevCon were controversial, but business-like and not too acerbic. The conference on the Establishment of a Middle East Zone Free of Nuclear Weapons and Other Weapons of Mass Destruction held in fall 2019 went relatively well. On the other hand, the NWS still refuse to even mention the TPNW, insist that it has a damaging impact on the NPT (NATO 2017; Gibbons 2019), and have exerted needless and futile pressure to prevent less powerful states from ratifying the ban treaty. Pro-TPNW NGOs, led by ICAN, are conducting a campaign aiming at undermining support for NATO’s extended deterrence arrangements, while non-democratic NWS, such as Russia or China, are largely immune to the political tools of this campaign.^7^ Repeated allegations that the TPNW entering into force will immediately make nuclear weapons “completely illegal” (e.g. Fihn and Thurlow 2017; ICAN 2020) feed the lack of trust that opponents of the TPNW have in the sincerity of the other side. Suggestions to “ditch” the NPT (Pretorius and Sauer 2019, 2021) cause concerns about the incompatibility of the two treaties.^8^

The TPNW is the result rather than the source of conflict in the NPT. Hence, discussions about the treaty further highlight the divisions that already existed within the NPT community, particularly over the right approach to disarmament (step-by-step vs. prohibitionist strategies) or about the political conditionality (or lack thereof) of disarmament undertakings. The TPNW, in particular, is thus a symbol of the deadlock between the demands of many NNWS for “tangible” disarmament, which goes beyond singular disarmament events, and the step-by-step approach to disarmament as propagated by the NWS and the NNWS allied to them. Disputes among the members of the NPT are now centered almost exclusively on the role, legitimacy, and desirability of the TPNW. Cleavages within the NPT community have, to some extent, become part of the debate on the ban treaty. This thematically narrow approach has only increased the polarization within the NPT. The question of whether the TPNW should be supported or condemned has turned into a question of faith (Müller and Wunderlich 2020), true to the motto: “whoever is not with me is against me”. The reflex logic of either categorically condemning or supporting the TPNW has led to a further neutralization of middle positions, marginalization of mediating strategies, and suppression of seemingly “uncontroversial” topics with an important consequence. Treating questions of nuclear disarmament as a matter of faith prevents a deeper debate on the opportunities but also on the problems of the TPNW.

As the TPNW is the result of pre-existing cleavages among NPT parties, exacerbated by the lack of, or even reversal of, progress in disarmament, it is obvious that the non-proliferation regime would be in trouble even if the TPNW did not exist. To unlock the stalemate and to get back on track, the “magic trigger” would be some tangible progress on the disarmament front. In this sense, the key is very much in the hands of the NWS.

### Nuclear proliferation in East Asia and the Middle East: A crisis of compliance and enforcement

The NPT has been rightly praised for having effectively curbed the spread of nuclear weapons, thereby establishing a strong non-proliferation norm (Rublee 2008; Carranza 2019). Instead of the dawning of a new nuclear age envisioned by President John F. Kennedy in 1963,^9^ today, only nine states possess nuclear weapons. Besides the five official nuclear weapon states as recognized by the NPT, India, Pakistan, and Israel have developed nuclear weapon capabilities outside the treaty regime. The broad majority of states has either remained or decided to become non-nuclear.

While most past cases of non-compliance have been effectively dealt with by treaty instruments, currently three proliferation cases are a cause for concern^10^: Following its withdrawal from the NPT in 2003, North Korea has steadily improved its military nuclear and missile capabilities. The case of serious safeguards breaches by Syria is still pending, while Iran at the very least made steady progress toward a nuclear weapons capability up until 2003. Not only are these cases problematic in themselves, as they increase the probability of nuclear weapons use. They also illustrate what might be considered the twofold Achilles heel of the nuclear non-proliferation regime—an inherently weak compliance and enforcement system that is also subject to the political will of the member states and highly dependent on cordial great power relations (Wunderlich et al. 2021, p. 22).

Ultimately, all three cases illustrate and are direct consequences of the NPT’s compliance and enforcement provisions, which can only work when the P5 support them unanimously. Verification activities by the IAEA remain limited when the country in question is neither applying an AP nor willing to cooperate with the Agency. This is most obvious in the case of the Democratic People’s Republic of Korea (DPRK). Under pressure from the Soviet Union, Pyongyang joined the NPT in 1985. That it took the country more than six years to conclude a comprehensive safeguards agreement (CSA) with the IAEA already raised suspicions as to the country’s compliance, which could, however, not be addressed because the NPT’s compliance/enforcement mechanism (Findlay 2015, p. 39) depends on having a safeguards agreement in force. When in 1992, North Korea ultimately concluded a CSA with the IAEA, initial inspections indeed revealed a secret plutonium program. Pyongyang refused to engage in any efforts to clarify the issue and, instead of cooperating with the Agency, announced its intention to withdraw from the NPT in 1993. While Pyongyang was persuaded to “suspend” this decision, it continued to obstruct verification work by the Agency in the years that followed. The IAEA was never able to fully resolve outstanding non-compliance concerns and when the United States confronted North Korea with evidence of an undeclared uranium enrichment facility, Pyongyang withdrew from the NPT in 2003. IAEA on-site verification and monitoring activities restarted in 2007, only to be restricted again in 2008 and completely stopped by April 2009. Since then, the IAEA has only carried out select (remote) activities. In 2018, a diplomatic rapprochement between the US and the DPRK resulted in an official North Korean commitment to denuclearization and freezes of nuclear activities (The White House 2018) which collapsed in 2019. Since then, North Korea has not undertaken any further nuclear weapon tests but has continued to develop delivery vehicles. The case of North Korea has not only triggered a debate as to the legality of the withdrawal decision (see, e.g. Bunn and Timerbaev 2005), but has also led to recurring disputes at RevCons on whether (and if so, how) to strengthen Article X of the NPT. Some actors (Canada, Germany, the EU) sought to qualify the right to withdrawal so that parties cannot misuse it first by cheating and then by leaving the NPT with an almost complete nuclear weapons program developed under the pretext of peaceful uses. But all these attempts have met with resistance from various sources and ultimately failed.

The Syrian proliferation case is also grounded in the dependence of the NPT’s compliance and enforcement system on P5 agreement and support. Suspicions were raised when the Israeli Air Force destroyed an alleged plutonium production reactor complex in 2007, which Syria had allegedly built with the help of North Korea but not reported to the IAEA. Ensuing inspections found evidence of undeclared activities, leading to a report by the IAEA Board of Governors affirming Syrian non-compliance with its safeguards obligations. Even while Syria indicated its willingness to accept inspections at the suspected site, after it had been cleaned, Damascus refused to allow investigations at other locations. Since then there has been no progress on the matter. Because Syria has not concluded an AP with the IAEA, the Agency is only able to verify the accuracy of inventories of declared nuclear materials, but not the absence of undeclared nuclear activities (Heinonen 2020, p. 23).

The third ongoing case of proliferation concern is also characterized by the fact that, for decades, Iran violated its safeguards obligations in order to acquire nuclear weapons capability at least up until 2003 (for the following, see Findlay 2015, p. 50–67; Heinonen 2020, p. 17–19). Revelations in 2002 that Iran had secretly built enrichment and reprocessing facilities led to intensified verification and inspection activities by the IAEA and diplomatic efforts outside formal treaty structures to solve the issue. While the Agency succeeded in persuading Iran to apply the AP in 2003, over the years the country oscillated between cooperative (enhanced transparency, suspension of sensitive activities) and confrontational behavior (denial of information, delaying tactics, resumption of suspended activities, construction of new facilities). From 2006 on, Iran was subjected to a strict sanctions regime, which hit the country’s economy hard. In 2015, changes in government in Teheran and Washington eventually led to a breakthrough in negotiations between the E3/EU+3 (France, Germany, the United Kingdom, the EU Representative for Foreign and Security Policy, the US, China, and Russia) and Iran. The Joint Comprehensive Plan of Action (JCPoA) severely constrained Iran’s enrichment and reprocessing activities and resulted in the country being subject to an unprecedented verification and inspection regime overseen by the IAEA. In return, Iran was granted access to support for peaceful nuclear activities and a gradual lifting of sanctions.^11^ While the case reveals the weakness of the NPT’s compliance and enforcement system, over the years it has also led to a strengthening of the inspection and monitoring capacities of the IAEA and further highlighted the value of the AP (Meier 2014). It is not without reason that the JCPoA has been referred to as the “new gold standard for non-proliferation agreements” (Pickering 2017).

The Iran case also illustrates the dependence on the political will of the five nuclear weapon states (and permanent members of the UNSC) when it comes to effectively dealing with non-proliferation cases under the NPT. The lack of favorable political environment—i.e. great power cooperation—makes it difficult to enforce norms (Müller 2000). While the JCPoA has been praised as a “net plus for international nuclear nonproliferation efforts”^12^ and Iran had repeatedly proven that it has met the terms of the IAEA agreement, the JCPoA has fallen victim to shortsighted geopolitical interests and the domestic concerns of ultraconservative US Republicans. In 2018, the Trump administration withdrew from the agreement and reimposed unilateral sanctions against Iran. In response, from May 2019 on, Iran gradually began to withdraw from its commitments under the JCPoA, including by resuming uranium enrichment, research and development on advanced centrifuges, and the expansion of its stockpile of nuclear fuel (Fitzpatrick 2020). In January 2020, Iran announced that it no longer felt bound by the limits set by the agreement, but that it would adhere to its safeguards obligations. When the E3 triggered the treaty’s dispute resolution mechanism to resolve the issue, Iran threatened to withdraw from the NPT (Panda 2020). This ultimately did not happen, and Iran has refrained—for the time being—from further violations of its obligations under the JCPoA. The Trump administration continued its strategy of maximum pressure and attempted to reimpose UN sanctions on Iran by using the “snapback” mechanism that is built into the JCPoA. This move was rejected by the vast majority of UNSC members, who argued that the US was no longer a participant in the agreement due to its withdrawal and therefore had no legal right to trigger a mechanism that was only available to JCPoA parties.^13^ While a solution to the crisis seemed impossible under Trump, the election of Joe Biden brought new hopes for salvaging the nuclear accord. At the time of writing, tensions still prevail, however. While Washington has changed its diplomatic course and no longer ties the resumption of talks to Iran’s return to compliance with the agreement, sanctions remain in place. Iran, meanwhile, continues to engage in nuclear brinkmanship, including further violations of its JCPoA obligations. In February 2021, it even suspended the implementation of the Additional Protocol and other JCPoA verification measures that go beyond its NPT safeguards agreement. The election of a well-known hardliner as president dampens any remaining optimism. With the conclusion of an interim monitoring agreement between the IAEA and Iran, the window for a diplomatic solution is still open (Associated Press 2021). But the case aptly illustrates how the Trump administration’s course of action has undermined what had been reasonably successful control of Iranian nuclear activities.

Great power interference has also hindered norm enforcement in the Syrian and North Korean proliferation cases. Russia has prevented a thorough investigation of Syria’s nuclear activities after the Israeli Air Force bombed a reactor that was under construction. Not only did China cast a negative vote when the IAEA found North Korea to be in non-compliance with its safeguards obligations in April 2003 and referred the case to the UNSC. It was also due to Chinese influence that the language of the resolution that was finally adopted by the Council, as well as all the subsequent resolutions, remained rather weak. Neither UNSC sanctions, nor the Six-Party-talks (DPRK, USA, China, Russia, Japan, and South Korea) initiated in 2003 could prevent North Korea from steadily developing its nuclear capabilities. Its first nuclear test in 2006 was followed by further tests in 2009, 2013, 2016, and 2017. China agreed to some sanctions against North Korea after each of the tests, but prevented sanctions that would have been harsh enough to make Pyongyang reconsider its course.

These unresolved proliferation cases could contribute to drive further proliferation. Nowhere is this more visible than in the Middle East. In addition to Iran, whose attempt to at least significantly delay the achievement of whatever military nuclear ambitions exist was destroyed by the Trump administration’s irreconcilable hostility, Syria, Turkey, and Saudi Arabia have given practical or verbal notice of their conditional readiness to abandon the virtuous path of non-proliferation. It remains to be seen in which direction the nuclear energy program of the VAE will turn. A flicker of nuclear ambition is also visible among America’s East Asian allies.

## Restabilizing the nuclear non-proliferation regime

In this section, we seek to outline the conditions for a restabilization of the non-proliferation regime and to draw the boundaries of a possible area of agreement between both sides of what is currently a deep divide. This does not necessarily mean that these conditions will (or can) be met, as this depends on the contingencies of the negotiation process and the further development of the antagonists’ positions. This chapter is therefore not intended to be a strategic prescription (which is a matter for campaigners and diplomats) or a blueprint for an agreement (the specifics of which entirely depend on the negotiating parties and the dynamics of their interaction). Rather, the focus is on determining where windows of opportunity may exist. Whether or not these opportunities will be seized depends on the actors on the ground.

Three changes are needed to restabilize the non-proliferation regime: First and most importantly, the ongoing power struggle between the great powers needs to be settled in a cooperative manner, including the nuclear dimension and the initiation of a process of plurilateral arms control and disarmament. This process has to emanate from the two states that possess the largest arsenals, the US and Russia. The decision to extend the New START Treaty for five years is an important first step as it reinstates much needed provisions for transparency and fostering confidence-building norms. Such a process should also aim to gradually include the other nuclear weapon states as recognized by the NPT and eventually, the countries possessing nuclear weapons outside the regime. While specific regional solutions are likely to be needed for the cases of Israel and North Korea, the P5 process appears to be an appropriate forum for sustained engagement with India and Pakistan, particularly with regard to working toward a mutual understanding of non-proliferation and disarmament priorities and discussing risk reduction measures. A unified approach is also needed to deal with regional challenges to non-proliferation. Another development that is indispensable for a cooperative settlement of current power struggles is that the great powers overcome their quest for narrow geopolitical interests and acknowledge their responsibilities when it comes to upholding international security (Leveringhaus and Hurrell 2018).

Second, the differences between the various groupings in the NPT need to be overcome (Miller 2012). This is particularly urgent with regard to the emotionally charged polarization on the issue of nuclear disarmament. Both sides, staunch proponents of a nuclear ban, on the one hand, and the NWS and their allies, on the other, must abandon their overly rigid positions and move toward each other, respecting the position of the other side and recognizing that their respective counter-positions are compatible with the NPT and nuclear disarmament. Such progress is hindered by the positions of some actors on both sides of the frontline and the absence of credible mediators—both factors limit the prospects of a return to political reason (Müller and Wunderlich 2020). It is therefore necessary for both supporters and opponents of the TPNW, civil society actors and governments, NWS and NNWS, to refocus on common objectives (preventing the use and proliferation of nuclear weapons, halting the arms race, achieving nuclear disarmament) and resume dialogue on meaningful steps to eliminate nuclear weapons. In practical terms, this could mean reaffirming the compatibility of the NPT and the TPNW and stressing common themes, such as the catastrophic humanitarian impacts of nuclear weapons. Ideally, the NNWS allied to the nuclear powers could play an even more constructive role by participating as observers in the first meeting of states parties to the TPNW in March 2022.

Third, it is of utmost importance that the principle of the prevention of nuclear war once again becomes the guiding principle behind the actions of NWS and NNWS. This step may pave the way for further changes in the direction of a peaceful regulation of the nuclear order. There are many possible ways of acknowledging this principle. Suggestions for responsibility-based approaches (Lodgaard 2020), pursuing the overarching normative goal of avoiding nuclear war, include the adoption of codes of conduct by the nuclear armed states (Gower 2019), “common but differentiated nuclear responsibilities” (Brixey-Williams 2019) of NWS and NNWS alike, “an institutionalized arrangement for monitoring and alleviation of risks” (Lodgaard 2019), and a “global regime of nuclear restraint and responsibility” (Tannenwald 2020, p. 206). While some of these proposals seem to be far-fetched given the current circumstances, the nuclear weapon states have repeatedly been urged to jointly reaffirm the 1985 Reagan-Gorbachev formula that “a nuclear war cannot be won and must never be fought”. The principle has been endorsed by various high representatives of the UN (e.g. the UN Secretary-General Guterres, UNODA 2018) and even China has (unsuccessfully) proposed that the principle be reaffirmed in the context of the P5 process, as well as in the NPT review cycle. While the US and Russia finally reiterated the formula in July 2021, reaffirming the clear avoidance of nuclear war must continue to be the focus of efforts by all nuclear weapon states. Lewis Dunn and William Potter (2020) have put forward a range of possibilities as to how state parties and civil society actors could seek to endorse the principle at the next NPT RevCon.

## Conclusion

Now, 50 years since it entered into force, the future of the NPT is more uncertain than ever. Temporary optimism about progress in disarmament has given way to disillusionment. Yet the destabilization of the NPT cannot simply be blamed on ban treaty enthusiasts nor on the geostrategic situation and looming great power conflicts. The growing rivalry between the US, Russia, and, more recently, China makes progress on disarmament increasingly unrealistic. Instances of nuclear proliferation put both the interplay between the great powers and the efficacy of the NPT’s compliance and enforcement mechanisms to the test. All this further exacerbates the deep divisions within the NPT community. The dichotomies within the NPT regime are evident not only when it comes to the question of how to handle the TPNW. However, finding the right approach to dealing with the TPNW could prove crucial for the future of the NPT. The TPNW is a direct product of the NPT’s shortcomings: the hierarchical structure of the NPT, the unequal treatment of its parties, and the disputes on the correct interpretation of the NPT’s norms have fueled the conflicts in the NPT community. Above all, it was the deep dissatisfaction of a large number of NNWS with the disarmament record of the NWS that gave rise to the TPNW. The response of the NNWS—by means of the TPNW—is a powerful normative comment on the state of nuclear disarmament as well as the NPT. Yet, the TPNW alone cannot guarantee sufficient security in a world without nuclear weapons. Nor will it lead to nuclear disarmament without the willingness and cooperation of the NWS.

The competition between the TPNW and the NPT, brought about by the misguided policies of the proponents of either side, has real-world consequences for the relationship between the NWS and the NNWS and the stability of the whole NPT regime. A further destabilization of multilateral nuclear arms control could exacerbate smoldering proliferation crises and reinforce the nuclear component of regional conflicts in the Middle East and East Asia alike. A crack in the already fragile foundation of the NPT would also affect ongoing attempts to rescue or revive bilateral nuclear arms control. Multilateral and bilateral nuclear arms control and disarmament are closely interwoven; the end of one can all too easily entail the end of the other. The entwinement of bilateral and multilateral arms control and disarmament is, in turn, the best chance we have of reducing the risk of nuclear war. A stabilization of the NPT and a channeling of the great power conflict into more cooperative forms could pave the way for nuclear disarmament more generally.

Given the current geostrategic situation, a cooperative process of plurilateral reconciliation hardly seems possible. Such a process would have to emanate from the United States and Russia; yet neither state seems willing to take the first step. As a consequence, other NWS, with China leading the way in pursuit of its own geopolitical agenda, can all too easily point to the nuclear superpowers’ failures in order to mask their own shortcomings. A reconciliation process jointly directed by the two nuclear superpowers would also help the NPT to overcome its extreme polarization. Yet, to mitigate the differences between the various groups within the NPT, the debate would have to be *de-emotionalized* while reminding all groups that no one wins if the regime fails. After all, “from its inception, the NPT was more than a body of international law. It was a political settlement among disparate states, and a statement of the values, norms and aspirations that should henceforth guide their behaviour” (Walker 2012). Reinvigorating the political energy of “middle powers” willing to comprise would also improve the situation. The most promising way of renewing support for the NPT would, however, be to return to the principle of prevention of nuclear war as a central goal and maxim for action. As long as fierce great power rivalry continues to exist, however, even this moderate idea seems out of reach.

## Footnotes

1 In line with the conceptualization presented in the introduction to this special issue (Kühn and Wisotzki, in this issue), for us the notion of “crisis” encompasses a material dimension as much as it represents a social construct that might be used as a rhetorical device.

2 For an account of the order’s normative basis, see Müller and Wunderlich (2020). For other conceptions of the nuclear order, see e.g. Biswas (2014); Ritchie (2019); Tannenwald (2018); Walker (2012).

3 The treaty text is available at https://www.un.org/disarmament/wmd/nuclear/npt/text/.

4 *United Nations* 2020: Security Council Press Statement on Supporting Non-Proliferation Treaty Ahead of 2020 Review Conference. Available at https://www.un.org/press/en/2020/sc14126.doc.htm, 10/21/2020.

5 We have not enumerated all possibilities here. The final documents of the NPT Review Conferences of 2000 (https://www.un.org/disarmament/wmd/nuclear/npt2000/final-documents/) and 2010 (https://www.un.org/en/conf/npt/2010/), as well as numerous non-governmental publications, offer a long menu of options. De-alerting steps, reductions in the biggest arsenals, capping the smaller arsenals, halting modernization programs, removing nuclear weapons from other states’ territories, and the new focus of the civil society campaign—no-first use—may be some of the top candidates. But it is up to the actors to choose. The remainder of this text focuses on the broader conditions for restabilizing the non-proliferation regime.

6 For a comprehensive overview of arguments put forward against the TPNW, see Müller and Wunderlich (2020).

7 On the philosophy and practice of the campaign, see Acheson (2018); Sauer and Reveraert (2018).

8 “Ditching” the NPT, that is, TPNW states parties leaving the NPT in large numbers with the objective of making the TPNW the only legal basis for the (non-)nuclear order, would, according to the protagonists of this idea, not damage the non-proliferation regime. This assessment, unfortunately, rests on incomplete and insufficient legal analysis. The invalidation of the NPT would not only mean that the rules for nuclear-related exports would disappear, but it would also immediately invalidate the safeguards agreements of these states with the IAEA, thus terminating verification. These agreements refer explicitly to the NPT as the basis in international law. Without this basis, safeguards agreements would lose validity and states could effectively refuse inspections. To address this legal deficit, there would have to be a new model safeguards agreement which refers to the TPNW. This sounds like a small thing, but in the treaty-making world, it requires rather a lot of effort. Such an agreement would have to be created against feasible attempts to adapt verification measures to the requirements of the TPNW (for which safeguards agreement INFCIRC/153 is insufficient), and be adopted by the IAEA Board of Governors (BoG) where non-parties to the TPNW are in the majority. Validation of the agreement for any single TPNW state would need new ratification by this state, which, based on previous experience, might take years. And it is also worth remembering that existing compliance and enforcement measures also hinge on the safeguards agreements.

9 Kennedy feared that by the 1970s, up to 25 states might have obtained military nuclear capabilities; see *Kennedy, John F.* 1963: “The President’s News Conference”, March 21, 1963. Available at https://www.presidency.ucsb.edu/documents/the-presidents-news-conference-169, 10/21/2020.

10 A detailed discussion of these cases can be found in Findlay (2015) and Heinonen (2020). Our account of the cases draws on these analyses.

11 However, Iran is still blocking investigations into past weaponization activities (Fitzpatrick 2020).

12 Statement from Nuclear Nonproliferation Specialists on the Iran Nuclear Deal, September 2017. Available at https://www.armscontrol.org/pressroom/2017-09/more-80-nuclear-nonproliferation-experts-reaffirm-support-iran-nuclear-deal, 10/21/2020.

13 See for instance the statement of the Foreign Ministers of Germany, France, and the United Kingdom, September 2020. Available at https://www.auswaertiges-amt.de/en/newsroom/news/-/2386144, Accessed: 23 Dez 2021.
